# Effects of Nitrogen Availability and Form on Phytoplankton Growth in a Eutrophied Estuary (Neuse River Estuary, NC, USA)

**DOI:** 10.1371/journal.pone.0160663

**Published:** 2016-08-09

**Authors:** Emily K. Cira, Hans W. Paerl, Michael S. Wetz

**Affiliations:** 1 Department of Physical and Environmental Sciences, Texas A and M University–Corpus Christi, Corpus Christi, Texas, United States of America; 2 Institute of Marine Sciences, University of North Carolina at Chapel Hill, Morehead City, North Carolina, United States of America; 3 Department of Life Sciences, Texas A and M University–Corpus Christi, Corpus Christi, Texas, United States of America; Stony Brook University, UNITED STATES

## Abstract

Nitrogen availability and form are important controls on estuarine phytoplankton growth. This study experimentally determined the influence of urea and nitrate additions on phytoplankton growth throughout the growing season (March 2012, June 2011, August 2011) in a temperate, eutrophied estuary (Neuse River Estuary, North Carolina, USA). Photopigments (chlorophyll *a* and diagnostic photopigments: peridinin, fucoxanthin, alloxanthin, zeaxanthin, chlorophyll b) and microscopy-based cell counts were used as indicators of phytoplankton growth. In March, the phytoplankton community was dominated by *Gyrodinium instriatum* and only fucoxanthin-based growth rates were stimulated by nitrogen addition. The limited response to nitrogen suggests other factors may control phytoplankton growth and community composition in early spring. In June, inorganic nitrogen concentrations were low and stimulatory effects of both nitrogen forms were observed for chlorophyll *a*- and diagnostic photopigment-based growth rates. In contrast, cell counts showed that only cryptophyte and dinoflagellate (*Heterocapsa rotundata*) growth were stimulated. Responses of other photopigments may have been due to an increase in pigment per cell or growth of plankton too small to be counted with the microscopic methods used. Despite high nitrate concentrations in August, growth rates were elevated in response to urea and/or nitrate addition for all photopigments except peridinin. However, this response was not observed in cell counts, again suggesting that pigment-based growth responses may not always be indicative of a true community and/or taxa-specific growth response. This highlights the need to employ targeted microscopy-based cell enumeration concurrent with pigment-based technology to facilitate a more complete understanding of phytoplankton dynamics in estuarine systems. These results are consistent with previous studies showing the seasonal importance of nitrogen availability in estuaries, and also reflect taxa-specific responses nitrogen availability. Finally, this study demonstrates that under nitrogen-limiting conditions, the phytoplankton community and its various taxa are capable of using both urea and nitrate to support growth.

## Introduction

In temperate and subtropical estuaries worldwide, phytoplankton growth can be controlled by factors such as nutrient and light availability, residence time, and grazing among others (e.g., [1–3]). Nitrogen (N) availability commonly limits estuarine phytoplankton growth, as strong correlations between N supply and phytoplankton production have been noted in many estuaries (e.g., [4–6]). As N loads have increased worldwide over the past century, harmful symptoms of eutrophication such as excessive chlorophyll *a* levels and increasing prevalence of harmful algal blooms and microbial pathogens, as well as hypoxia, have become widespread (e.g., [7, 8]). In river-dominated estuaries, N loading typically occurs in pulses following rain events, and these events have been shown to modulate large-scale spatial and temporal patterns in phytoplankton community growth and taxonomic composition (e.g., [3, 9, 10]).

While overall N availability is significant from the standpoint of controlling phytoplankton growth in many estuaries, N form may influence phytoplankton community structure by favoring the growth of certain taxa over others [11]. It has been noted that the forms of N delivered to many estuaries have shifted to include a larger proportion of reduced N compounds, including urea [12]. As a major component of fertilizers, urea levels have increased sharply in some estuaries subjected to loadings from watershed agricultural activities [12]. While urea has been shown to be a major source of N supporting phytoplankton growth in a number of systems (e.g., [13, 14]), its role in shaping phytoplankton community composition remains unresolved because of a paucity of observational and experimental studies, with existing studies showing considerable variability in the response of different phytoplankton taxa to urea [15, 16]. Experimental work on a range of potentially harmful taxa (e.g., *Prorocentrum minimum*, *Pseudonitzschia delicatissima*) has demonstrated their ability to utilize organic nutrient forms [17, 18], and it has been hypothesized that urea may favor the growth of mixotrophic, and potentially harmful, phytoplankton taxa [19].

Here, we experimentally determined the influence of urea and nitrate additions on estuarine phytoplankton growth and taxonomic composition in the Neuse River Estuary, NC. Our objectives were to examine the effects of N availability and the relative influence of each N form during the growing season from early spring through late summer. Results from these experiments add to a limited body of knowledge on the role(s) these N forms play in determining estuarine phytoplankton growth and community composition.

## Materials and Methods

### Study site–Neuse River Estuary, North Carolina

The Neuse River Estuary (NRE) in eastern North Carolina is part of the Albemarle-Pamlico estuarine system, the second largest estuarine complex in the United States. The NRE is a long residence time (up to four months), lagoonal estuary. N loading is usually highest in late winter/spring when river discharge is high [20, 21], while in the summer inorganic N concentrations are typically low except for occasional pulses during hypoxic or storm events [13, 20, 22]. Nitrate is the dominant inorganic N form in the NRE, while maximum urea concentrations are ≤ 3 μM [13, 20]. During the summer months, phytoplankton growth is often N-limited in the NRE [4, 13]. Increasing N loads over the past few decades have led to significant acceleration of eutrophication, including numerous phytoplankton blooms and presence of harmful taxa [21, 23–25].

### Experimental design

Similar to other estuaries, phytoplankton blooms tend to form in a distinct zone in the NRE termed the chlorophyll *a* maximum (CMAX) [26–29]. Experiments were conducted using surface water collected from the CMAX throughout the growing season, on June 6, 2011 (35.03˚N, 76.97˚W), August 15, 2011 (35.14˚N, 77.05˚W), and March 12, 2012 (34.84˚N, 76.87˚W). These locations are not within private or protected waters and no permission was required for taking samples. This study did not involve any endangered or protected species.

For each of the three experiments, N availability was manipulated by adding 10 μM-N as either urea or potassium nitrate to experimental treatments, while controls had no N addition. Treatments were run in triplicate for each experiment. Experimental water was dispensed in pre-washed (with 10% HCl) and rinsed (4x with deionized water) 4 L high density polyethylene Cubitainers that were ~80% transparent to ambient photosynthetically active radiation (PAR). Cubitainers were incubated in an outdoor pond (~1 m deep) that was continuously flushed with water from adjacent Bogue Sound, thereby approximating in situ temperature and surface light conditions. Subsamples were taken from each Cubitainer at 24 and 48 hours, but data are presented showing the phytoplankton response after 24 hours because one or more key nutrients (phosphorus [P] in March; N and P in June; N in August) were depleted to below typical half-saturation levels for uptake by 24 hours (i.e., <0.1–0.2 μM P, <1–2 μM N; [30]; S1 Table). Thus community dynamics and changes after that timeframe would not necessarily reflect a response to the added nutrients. Effects of N addition on phytoplankton growth and community composition were determined primarily from changes in chlorophyll *a* and phytoplankton group diagnostic photopigments, with supporting information provided by microscopic cell counts.

### Light, nutrients, pigments, and cell abundances

Photosynthetically available radiation (PAR) was monitored continuously with a LI-COR 2pi PAR sensor located adjacent to the pond. At the beginning of each experiment and after the 24 hour incubation period, subsamples were collected for inorganic nutrients, total dissolved N (TDN), phytoplankton diagnostic photopigments and phytoplankton abundance. Nutrient (NO_3_^-^, NO_2_^-^, NH_4_^+^, PO_4_^-^) and TDN analyses were conducted with a Lachat QuickChem 8000 (Lachat Instruments) according to standard colorimetric methods (see [31] for details). Dissolved organic N (DON) was estimated as the difference between TDN and inorganic N. Pigment concentrations were quantified using a high performance liquid chromatography (HPLC) photodiode array spectrophotometry system, as described in Paerl et al. [32]. Chlorophyll *a* and diagnostic photopigments for the major phytoplankton taxonomic groups in the NRE were measured [33, 34], including: zeaxanthin (cyanobacteria), fucoxanthin (raphidophytes, *Karlodinium* sp., diatoms, haptophytes), peridinin (dinoflagellates), chlorophyll b (chlorophytes, prasinophytes), and alloxanthin (cryptophytes). Samples for cell counts were preserved with acid Lugols (3–4% final conc.) and stored in amber glass bottles in the dark until analysis. 5 mL of subsample was settled in Utermohl chambers for 3 hours, deemed appropriate based on chamber volume and height (see [35, 36]). Cell counts were conducted using an Olympus IX71 inverted microscope at 200x magnification. Cells >5 μm were identified to lowest taxonomic level possible, and > 600 cells were counted per sample to ensure accurate representation of the various taxa.

Net phytoplankton growth rates (d^-1^) were calculated for each treatment using photopigment concentrations and/or cell abundances at the start of each experiment and at 24 hours. Statistical significance was determined first by Analysis of Variance (ANOVA; α = 0.05), followed by Tukey’s Post Hoc tests (α = 0.05) conducted with R Studio [37]. Assumptions of normality and homoscedasticity were checked with the Shapiro-Wilk test (α = 0.01) and the Brown-Forsythe Levene-type test (α = 0.05), and were met for all analyses.

## Results

### Initial conditions

Meteorological conditions varied among experiments, however, both integrated PAR and surface water temperatures were similar between June and August (Table 1). Integrated PAR was approximately 2-fold higher in the summer months than in March, and surface water temperatures were higher in summer months as well (Table 1).

**Table 1:** Initial nutrient concentrations and physical conditions for each experiment.

Initial Conditions	March 2012	June 2011	August 2011
Nitrate + nitrite (μM-N)	12.8	0.5	11.6
Orthophosphate (μM-P)	0.3	0.4	3.7
Ammonium (μM-N)	0.8	1.6	1.8
Inorganic N:P	40.7	4.9	3.6
Dissolved organic nitrogen (μM-N)	26.1	21.1	21.5
Integrated 24-hour PAR (E/m^2^)	25	49	46
Salinity	6.4	7.1	4.8
Surface water temperature (°C)	13	28	29

Throughout the experiments, DON accounted for a majority of TDN (Table 1). Nitrate concentrations (as nitrate + nitrite) were highest in March (12.8 μM-N), while ammonium (0.8 μM-N) and orthophosphate concentrations were low (0.3 μM-P), and inorganic molar N:P was 40.7 at that time. In June, ammonium concentrations were higher than nitrate concentrations (1.6 μM-N and 0.5 μM-N, respectively), while orthophosphate concentrations were 0.4 μM-P. Inorganic molar N:P was 4.9, indicative of N-limited conditions (Table 1). In August, nitrate and ammonium concentrations were relatively high (11.6 μM-N and 1.8 μM-N, respectively), while orthophosphate concentrations were also high (3.7 μM-P), and inorganic molar N:P (3.6) once again indicated N-limited conditions (Table 1). Although silicate was not measured, silicate concentrations at adjacent water quality monitoring stations were >15 μM-Si in March, >86 μM-Si in June and >90 μM-Si in July (H. Paerl, unpubl. data; available upon request).

Bloom-level concentrations of chlorophyll *a* (defined here as > 30 μg L^-1^; see [20, 28, 32] for long-term data) were observed in March (32.8 μg L^-1^), with photopigments (peridinin, 8.8 μg L^-1^) and cell counts indicating dominance by the dinoflagellate *Gyrodinium instriatum* (Tables 2 and 3). High fucoxanthin concentrations (2.5 μg L^-1^) and diatom abundances were also noted (Tables 2 and 3). The dominant diatoms included *Leptocylindrus* sp., *Skeletonema* sp., *Cyclotella* sp., and *Chaetoceros* sp (Table 3). Chlorophyll *a* concentrations were lower in June (17.2 μg L^-1^). Relatively high peridinin (2.2 μg L^-1^) and zeaxanthin (3.2 μg L^-1^) concentrations indicated that the community was dominated by dinoflagellates and cyanobacteria (Table 2). Cell counts showed very high abundances of the dinoflagellate *Heterocapsa rotundata* (Table 3). No cyanobacteria in the detectable size range (>~5 μm) were observed, indicating that the high zeaxanthin concentration may have arisen from picocyanobacteria that were too small to count but are known to be abundant in the Neuse River Estuary during the spring-fall period [38]. Elevated abundances of cryptophytes and raphidophytes were also noted (Table 3). Lowest chlorophyll *a* levels (15.3 μg L^-1^) were observed in August (Table 2). Phytoplankton pigments showed no clear dominant functional groups (Table 2), though cell counts indicated high abundances of *Anabaena* sp., cryptophytes and chlorophytes (Table 3).

**Table 2:** Initial concentrations of diagnostic photopigments (μg L^-1^) for each experiment.

Photopigments	March 2012	June 2011	August 2011
Chlorophyll *a*	32.8	17.2	15.3
Alloxanthin	BDL	0.7	0.8
Chlorophyll b	0.5	0.7	1.7
Fucoxanthin	2.5	0.6	1.5
Peridinin	8.8	2.2	0.6
Zeaxanthin	0.4	3.2	1.0

BDL indicates below detection limits.

**Table 3:** Initial abundances and growth rates (μ ± SD) of the major phytoplankton taxa in each experiment.

			24-hr growth rates (μ ± SD)		
	Taxa	T_0_ Abundance (cells mL^-1^)	Control	Nitrate	Urea
March	*G*. *instriatum*	1602	-0.92 ± 0.51	-1.13 ± 0.56	-0.50 ± 0.58
	*Karlodinium* sp.	53	-0.07 ± 0.36	-0.08 ± 0.23	-0.04 ± 0.11
	*Skeletonema* sp.	150	0.43 ± 0.29	0.02 ± 0.77	-0.12 ± 0.14
	*Chaetoceros* sp.	41	0.87 ± 0.52	1.13 ± 0.36	1.08 ± 0.27
	*Leptocylindrus* sp.	101	0.35 ± 0.51	0.43 ± 0.34	0.60 ± 0.22
	*Cyclotella* sp.	46	0.41 ± 0.43	0.15 ± 0.36	0.38 ± 0.14
	Chlorophytes	59	0.47 ± 0.40	0.56 ± 0.08	0.58 ± 0.24
June	*H*. *rotundata*	3211	0.19 ± 0.06	0.33 ± 0.24	0.64 ± 0.15 *(*p* = 0.04)
	Cryptophytes	459	-0.17 ± 0.20	0.58 ± 0.01 * (*p* = 0.04)	0.32 ± 0.44
	Chlorophytes	119	-0.02 ± 0.13	-0.15 ± 0.14	0.04 ± 0.15
	Raphidophytes	186	0.05 ± 0.19	0.01 ± 0.17	-0.08 ± 0.01
	*Karlodinium* sp.	66	0.97 ± 0.12	0.83 ± 0.33	0.64 ± 0.37
August	Cryptophytes	720	0.94 ± 0.05	0.52 ± 0.45	0.92 ± 0.27
	Chlorophytes	320	0.63 ± 0.05	0.28 ± 0.28	0.58 ± 0.24
	*Euglena* sp.	203	0.24 ± 0.08	0.58 ± 0.13	0.79 ± 0.27 *(*p* = 0.02)
	Diatoms	96	0.53 ± 0.15	0.44 ± 0.21	0.40 ±0.20
	*Anabaena* sp.	925	-0.15 ± 0.39	0.01 ± 0.42	-0.40 ±0.59

Asterisk (*) indicates statistically significant difference in growth rates between a N treatment and the control. Significant *p*-values are given in parentheses.

### Phytoplankton response to N additions

**Fig 1:**
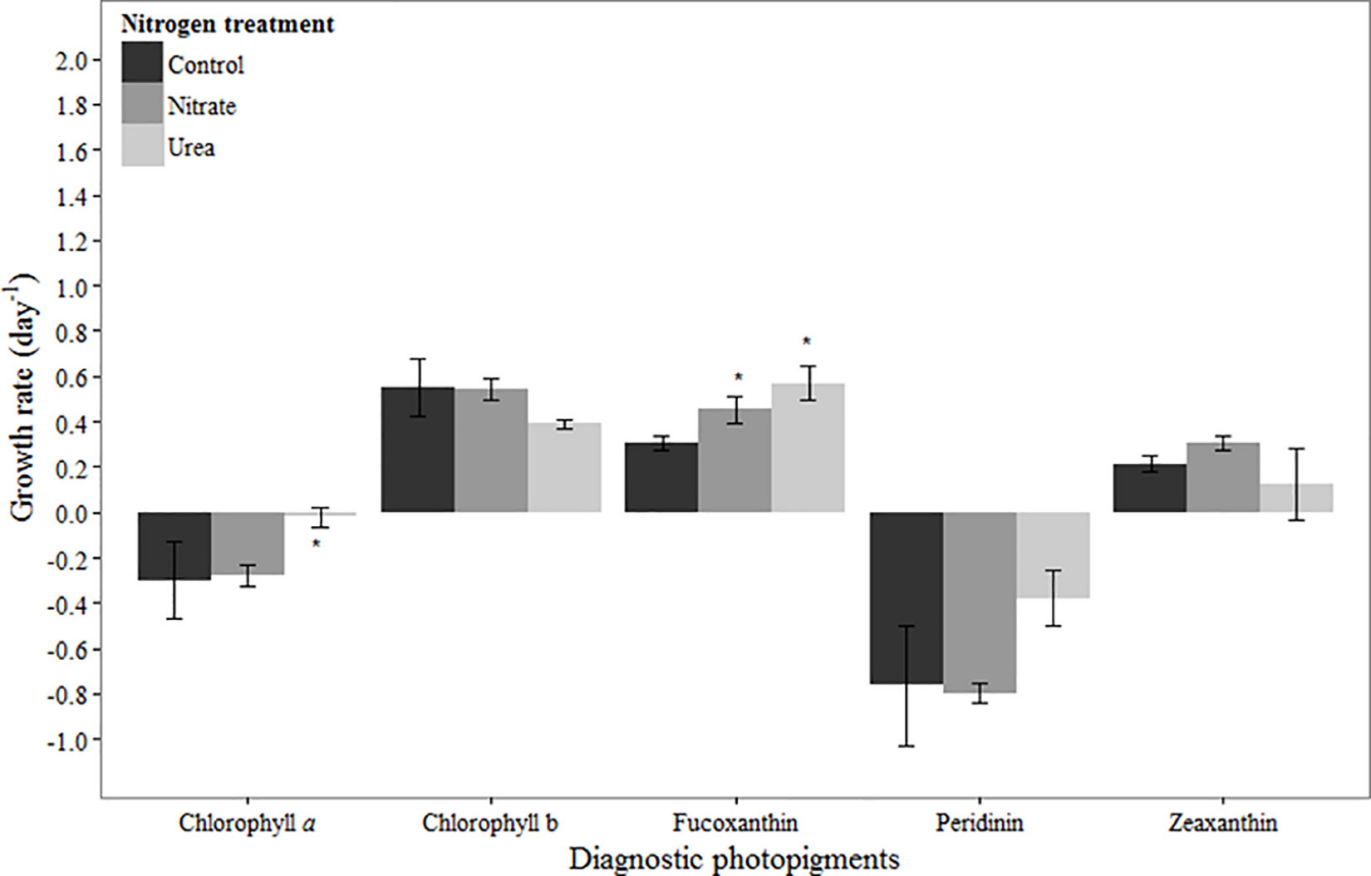
Growth rates based on diagnostic pigments in March 2012 experiment. Bars represent standard deviation (*n* = 3). Asterisk (*) indicates statistically significant difference between N treatment and control, while ‘a’ indicates statistically significant difference between nitrate and urea treatments. Refer to Table 2 for initial concentrations of pigments.

In March, chlorophyll *a*-based net growth rates were negative in all treatments, but higher in the urea addition treatment compared to the control (*p* = 0.04; Fig 1). The net growth rate in response to urea addition was higher than in response to nitrate, and this was statistically significant (*p* = 0.05; Fig 1). Chlorophyll b-, fucoxanthin- and zeaxanthin-based net growth rates were positive in all treatments (Fig 1). No difference was observed between the control and N treatments for chlorophyll b (urea: *p* = 0.10, nitrate: *p* = 0.99) and zeaxanthin (urea: *p* = 0.51, nitrate: *p* = 0.13). Likewise, no difference was observed between treatments for the dominant chlorophyll b-containing taxa that were enumerated, the chlorophytes (Table 3). In contrast, fucoxanthin-based net growth rates were significantly higher in the urea and nitrate treatments compared to the control treatment (*p <* 0.01, *p* = 0.04, respectively; Fig 1). For some diatom taxa, mean growth rates in N-additions were lower than in the control treatments (*Skeletonema* sp., *Cyclotella* sp.), while others (*Leptocylindrus* sp., *Chaetoceros* sp.) had higher net growth rates in both N amended treatments compared to the control (Table 3). However, differences in growth rates were not significant for any of these taxa. Another fucoxanthin-containing taxon, the dinoflagellate *Karlodinium* sp., did not respond to N addition (Table 3). Peridinin-based net growth rates were negative in all treatments (Fig 1). Peridinin-based growth was slightly higher in the urea treatment compared to the nitrate or control treatments, though the difference was not statistically significant (*p* = 0.06, p = 0.08, respectively; Fig 1). Net growth rates of the dominant dinoflagellate, *Gyrodinium instriatum*, were also negative in all treatments, and though statistically indistinguishable, the mean growth rate was highest in the urea addition treatment (Table 3).

**Fig 2:**
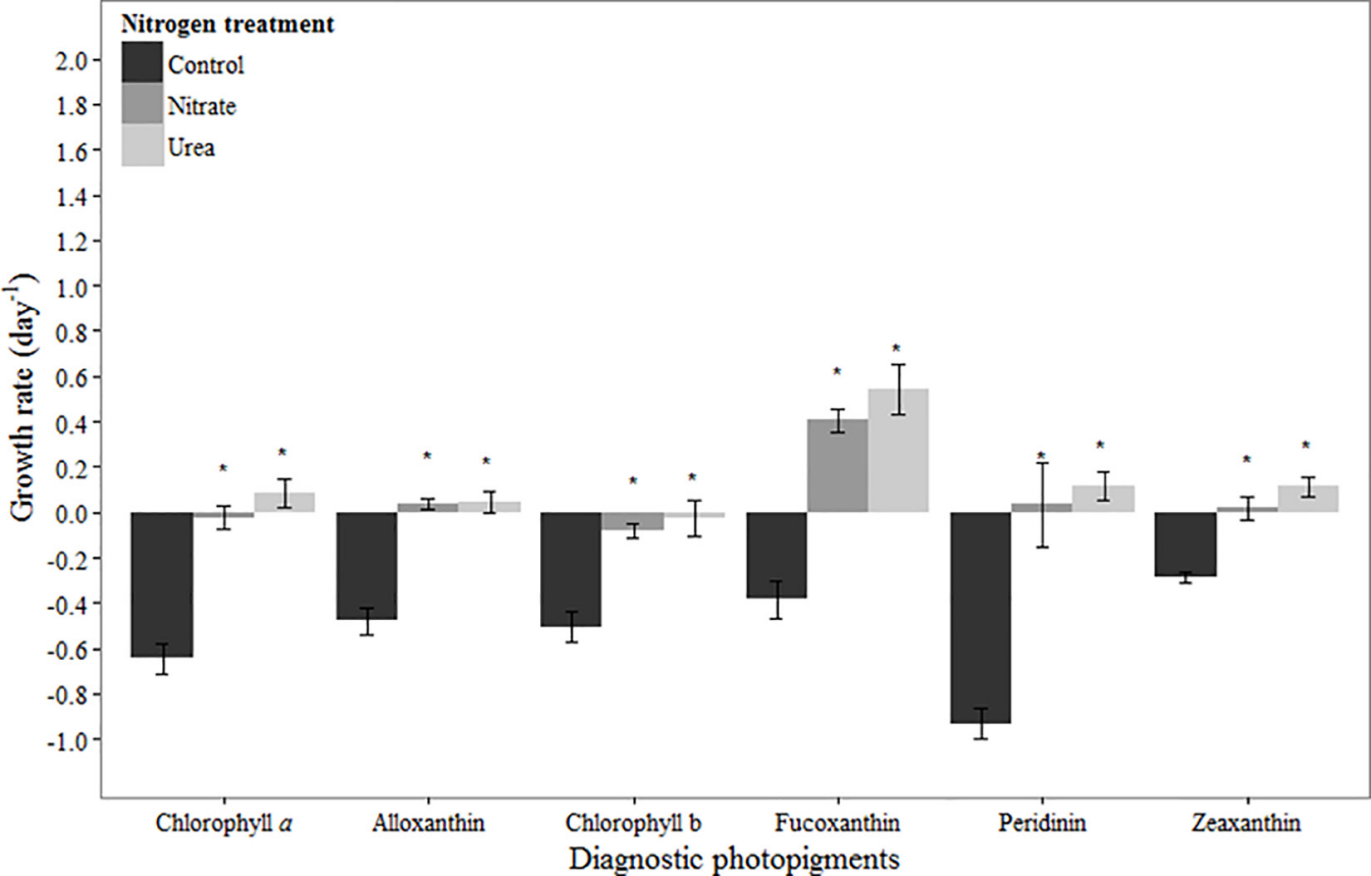
Growth rates based on diagnostic pigments in June 2011 experiment. Bars represent standard deviation (*n* = 3). Asterisk (*) indicates statistically significant difference between N treatment and control. Refer to Table 2 for initial concentrations of pigments.

In June, the chlorophyll *a-*based net growth rate was negative in the control, near zero in the nitrate treatment, and slightly positive in the urea treatment (Fig 2). Net growth rates in both N amended treatments were significantly higher than in the control treatment (urea: *p* < 0.001, nitrate: *p* < 0.001), but there was no significant difference between the N forms (*p* = 0.16; Fig 2). As with chlorophyll *a*, net growth rates based on each of the diagnostic photopigments were negative in control treatments (Fig 2), and were significantly higher in urea (all pigments: *p* < 0.001) and nitrate addition treatments compared to the control (all pigments: *p* < 0.001). No significant differences were observed between N forms for any of the pigments (Fig 2). Fucoxanthin displayed positive net growth rates of 0.4–0.6 d^-1^ in N addition treatments (Fig 2), whereas the net growth rates of the two main fucoxanthin-containing taxa, the raphidophytes and *Karlodinium* sp., were not stimulated by N addition (Table 3). Growth of the most abundant phytoplankton taxa, *Heterocapsa rotundata*, was positive in all treatments and was significantly higher in the urea addition treatment compared to the control treatment (Table 3; *p* = 0.04). Cryptophyte growth rates generally mirrored those of alloxanthin, with negative growth in control treatments and positive growth in both N treatments, though only in the nitrate addition treatment was growth significantly higher than that in the control (Table 3; *p* = 0.04). The only chlorophyll b-containing taxa enumerated, the chlorophytes, showed no obvious response to N addition compared to controls (Table 3).

**Fig 3:**
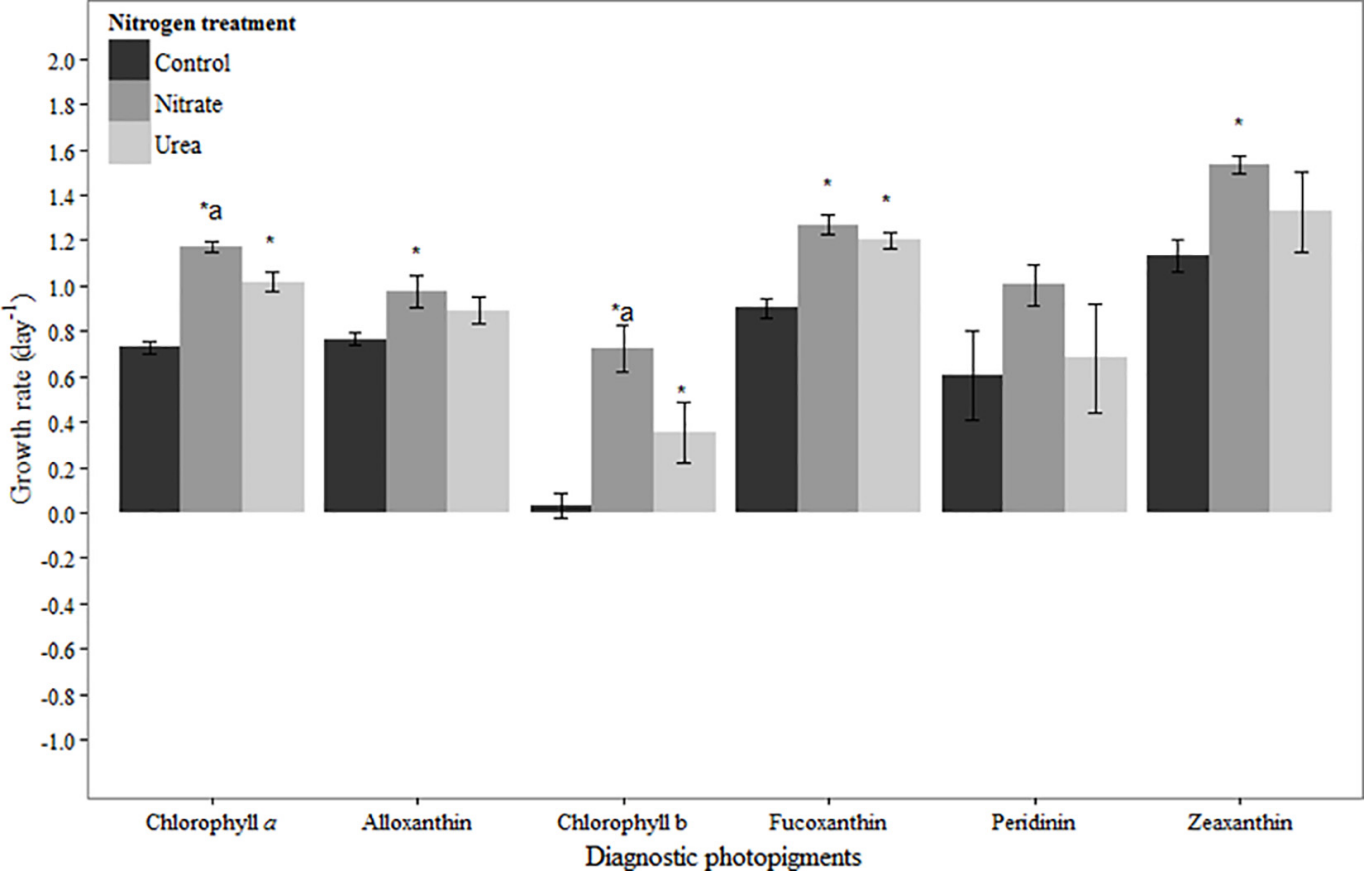
Growth rates based on diagnostic pigments in August 2011 experiment. Bars represent standard deviation (*n* = 3). Asterisk (*) indicates statistically significant difference between N treatment and control, while ‘a’ indicates statistically significant difference between nitrate and urea treatments. Refer to Table 2 for initial concentrations of pigments.

In August, chlorophyll *a*-based net growth rates were positive in the control and N amended treatments (Fig 3). Net growth rates were significantly higher in N addition treatments compared to the control (urea: *p* < 0.001, nitrate: *p* < 0.001), and significantly higher in the nitrate treatment compared to the urea treatment (*p* < 0.01; Fig 3). All diagnostic photopigments had positive net growth rates in the control and N amended treatments (Fig 3). Zeaxanthin- and alloxanthin-based net growth rates were significantly higher in the nitrate treatment compared to the control (*p* = 0.01, *p =* 0.01, respectively), but not in the urea treatment (*p* = 0.17, *p* = 0.07, respectively). The only zeaxanthin-containing taxa enumerated, *Anabaena* sp., showed no response to N addition (Table 3). Likewise, cryptophyte abundances did not indicate a stimulatory effect of N addition on growth (Table 3), contrary to the response of their marker pigment alloxanthin. Chlorophyll b-based net growth rates were significantly higher in N addition treatments compared to the control (urea: *p* = 0.02, nitrate: *p* < 0.001), and significantly higher in the nitrate treatment compared to the urea treatment (*p* = 0.01; Fig 3). Of the chlorophyll b-containing taxa enumerated, chlorophyte growth was not stimulated by N addition, whereas *Euglena* sp. growth was stimulated by urea addition (Table 3; *p* = 0.02) but not nitrate (Table 3). Fucoxanthin-based net growth rates were significantly higher in both N addition treatments relative to the control (urea: *p* < 0.001, nitrate: *p* < 0.001; Fig 3). However, growth of the only two fucoxanthin-containing taxa enumerated, diatoms and raphidophytes, was not stimulated by N addition (Table 3). No statistically significant effect of N addition was observed on peridinin-based net growth rates (Fig 3).

## Discussion

In these experiments, the phytoplankton responses to N additions and two different N forms were examined under representative seasonal conditions in the NRE, including; 1) cool, low light, early springtime conditions, 2) warm, low N summertime conditions, and 3) warm, N replete summertime conditions. Others have demonstrated distinct changes in both NRE phytoplankton growth and photopigment-based functional groups in response to these various conditions (e.g., [34]). As much as feasible, this study went further by examining the ecological response of not only broad phytoplankton functional groups but also specific phytoplankton taxa to N addition, yielding new information on phytoplankton taxa that in many cases are important not only in the NRE but also other temperate/subtropical systems.

### March

In March, a bloom of the dinoflagellate *G*. *instriatum* was in place at the initiation of our experiment. Time course changes in its marker photopigment peridinin were similar to those of chlorophyll *a*, suggesting that the bulk of chlorophyll *a* present was from *G*. *instriatum*. *G*. *instriatum* abundances and peridinin concentrations were both higher in the urea treatment compared to the control and nitrate treatments after 24 hours (Fig 2, Table 3). While Nagasoe et al. [39] determined that one particular strain of *G*. *instriatum* was unable to use urea and other organic N compounds for growth, results presented here suggest that this organism has the ability to use urea and/or was stimulated by growth of prey organisms that it is known to phagocytize (e.g., [40]). Of the other pigments observed in March, chlorophyll b- and zeaxanthin-based net growth rates were positive and unaffected by N availability. Fucoxanthin-based net growth rates were positive in all treatments, but significantly higher in N treatments compared to the control, suggesting suboptimal growth at ambient concentrations and overall N limitation of growth. Furthermore, the stimulatory effects of nitrate and urea were equivalent, underscoring the ability of diatoms to utilize urea as an N source [15, 41]. Rothenberger et al. [25] have argued that the relative importance of diatoms compared to dinoflagellates in the NRE during late winter-spring is dictated largely by river flow, with diatoms favored during high flow, elevated nutrient loading conditions. Our results are consistent with this, showing a much stronger response to N addition by diatoms compared to dinoflagellates based on pigment data. However, the diatom response was genera-specific, with some diatom genera being stimulated by N and others not. This points to effects of N availability that extend beyond broad functional group classifications to the genera or species level.

Results indicate that the influence of N can be modulated by, or eclipsed by, other factors during late winter/early spring in the NRE. For example, the low light levels in the turbid NRE may ultimately drive the community to N-limitation by preventing nitrate uptake, particularly in the late winter/early spring months. In this experiment, even though nitrate was present at a moderately high concentration in the environment (12.8 μM-N), chlorophyll *a*-based net growth rates in the control as well as nitrate treatments were negative. Additionally, the chlorophyll *a*-based growth rate was highest in the urea addition treatment, suggesting that urea, not nitrate, was more effective at supporting community growth in March. Reduced N forms such as urea have been shown to be energetically favorable over nitrate under relatively low light conditions such as those experienced in mid-March [42, 43]. Another factor that can account for the lack of response to N additions in March is P-limitation or co-limitation. At the initiation of this experiment, phosphate concentrations were low and inorganic molar N:P was ~41, suggesting that P-limitation may have prevented a stimulatory effect of N additions. Co-limitation of phytoplankton growth by N and P has been noted in spring in the NRE [4]. Aside from that of some diatom genera, the weak response of the phytoplankton community to N additions in March stresses the importance of other regulating/limiting factors in bloom dynamics during this time of year.

### June

In June, the negative growth response in controls for all pigments, coincident with very low inorganic N concentrations, is indicative of strong N-limitation. Furthermore, chlorophyll *a*-based net growth rates as well as those of the main diagnostic pigments (alloxanthin, chlorophyll b, fucoxanthin, peridinin, zeaxanthin) were stimulated by N addition, with no significant differences between forms. In the case of alloxanthin and peridinin, net growth rates of the dominant taxa represented by these two pigments (cryptophytes and *Heterocapsa rotundata*, respectively) showed that the pigment-based growth changes were indeed representative of actual cell growth (Fig 2; Table 3). Cryptophytes often produce some of the largest blooms in the NRE, usually following nutrient pulses into the system [33]. Although some cryptophytes are mixotrophic [17, 44–46], few studies have demonstrated a clear preference for DIN or organic N compounds (e.g., [16]). Cryptophyte growth in June was significantly stimulated by nitrate addition, while the response to urea addition was lower and more variable (Table 3), suggesting potential preference for DIN under the environmental conditions of the June experiment. *H*. *rotundata* was the dominant dinoflagellate in June and has been shown to form blooms in other estuarine systems such as the Barnegat Bay-Little Egg Harbor system, NJ [47] and the Chesapeake Bay [48]. While *H*. *rotundata* responded significantly to urea addition in this experiment (Table 3), it has not been shown to directly utilize urea [49, 50]. This species, however, is often associated with high ammonium concentrations [25]. Thus, the response to urea additions may be driven by ammonium released through bacterial remineralization of the urea, which (bacterial remineralization) can be high in the summer months in the NRE [25, 51]. The mixotrophic capabilities of this numerically important microorganism merit further investigation.

In contrast to alloxanthin and peridinin, it is difficult to verify that increases in the other pigment concentrations in response to N addition were actually reflective of increased cell growth. For example, because no zeaxanthin-containing organisms were detected in our cell counts that were limited to >5 μm cells, it can only be assumed that the high zeaxanthin concentrations were indicative of picocyanobacteria (*Synechococcus* sp.), which are numerically very abundant during summer in the NRE [38] and are capable of using both inorganic and organic N in coastal settings (e.g., [52, 53]). Abundances of the main fucoxanthin- (*Karlodinium* sp., raphidophytes) and chlorophyll b- (chlorophytes) containing organisms did not increase in response to N addition. In fact, *Karlodinium* sp. had a relatively high net growth rate in all treatments (0.6–1.0 d^-1^), suggesting that its flexible nutritional strategies allowed it to thrive under the low inorganic N conditions in June (e.g., [54, 55]). Net growth rates of the chlorophytes and raphidophytes were very low, and did not respond to N additions, suggesting that they may have been under strong grazing control, which has been previously documented to occur in the NRE during summer [56].

There are two possible explanations for the phenomenon of a stimulatory effect of N addition on the marker pigments without a concomitant effect on cell abundances. One possibility is that we simply were unable to count the truly dominant taxa represented by fucoxanthin and chlorophyll b due to morphological ambiguities or small size, as is suspected for zeaxanthin-containing organisms (e.g., cyanobacterial picoplankton). Examples may include small haptophytes (fucoxanthin), which are known to be abundant in the NRE in summer/fall [25], and prasinophytes (chlorophyll b), which can also be important in the NRE under warmer conditions [25]. The second possibility is that the proportion of marker pigment per cell changed in response to N addition (e.g., [57, 58]). In this regard, Lewitus et al. [59] found that the fucoxanthin content per cell was generally higher in three estuarine flagellate species grown under nutrient replete versus deplete conditions. Both possibilities highlight the difficulty in interpreting ecological patterns and relationships among estuarine phytoplankton taxa from pigments without use of other complimentary approaches, such as microscopy.

### August

Chlorophyll *a-* and diagnostic pigment-based net growth rates were positive in August across all treatments. Despite this and the fact that nitrate concentrations were relatively high (Table 1), pigment-based net growth rates were stimulated by N addition in several cases. This may seemingly point to suboptimal phytoplankton growth under ambient conditions as a result of N limitation. However, there were very few examples in August where the pigment-based growth changes were mirrored by those of the dominant phytoplankton taxa as determined by microscopy. This suggests that either pigments captured the growth response of taxa that we were unable to enumerate (e.g., prasinophytes–chlorophyll b, haptophytes–fucoxanthin), or that changes in pigment concentrations reflected a change in cellular pigment levels. For example, cryptophyte growth was positive in all treatments, but was not stimulated by nitrate addition in contrast to its marker pigment, alloxanthin (Table 3, Fig 3). This suggests that the amount of alloxanthin per cell may have increased as a result of increased nitrate availability (cf. [59, 60]), and thus the increased alloxanthin concentration was not reflective of a true growth response to N addition. In the case of chlorophyll b-containing organisms, chlorophytes and *Euglena* sp. were the main taxa present and their growth was also positive in all treatments. Yet whereas the chlorophytes were not stimulated by N addition, *Euglena* sp. growth was stimulated by urea (Table 3). With these two taxa alone, it remains unclear to what extent the change in chlorophyll b was representative of a true growth response (primarily of *Euglena* sp.), changes in pigment per cell with no true growth response, or reflective of taxa (e.g., prasinophytes) that we were unable to enumerate.

Finally, growth of the main zeaxanthin-containing organism, *Anabaena* sp., was near zero to negative in all treatments in contrast to positive zeaxanthin-based growth rates (Table 3, Fig 3). *Anabaena* spp. is a numerically-important taxa in the upper NRE during summer-fall [25], and many species are known to be N_2_ fixers (e.g., [61]). Thus it is not surprising that it did not respond to N addition. While there may have been changes in per cell concentrations of zeaxanthin in *Anabaena* sp. over the course of this experiment, we suspect that picocyanobacteria were a significant contributor to zeaxanthin in August (cf. [38]). These results clearly demonstrate the importance of incorporating both pigment analysis and cell counts in studies of phytoplankton ecological dynamics. In this experiment, relying solely on either method would have led to vastly different conclusions about the magnitude of growth and the ecological effects of N addition on the NRE phytoplankton community and its important taxa.

## Conclusions

Results from this study show seasonal and taxa-specific variability in the influence of N availability on phytoplankton growth, as has been noted in the NRE and elsewhere [2, 4, 62]. Clear signs of N-limitation were seen in the summer months, while in the late winter/early spring, this was not the case, stressing the importance of co-occurring physical or chemical factors (e.g., low light levels, P-limitation) that may regulate bloom dynamics during that period. The observed effects of N addition on phytoplankton growth and community composition in these incubation experiments are broadly consistent with in situ seasonal dynamics in the NRE [25] and other coastal systems [16, 63, 64], and add to our understanding of the intricacies of the nitrogen-phytoplankton relationship for specific estuarine phytoplankton taxa.

Subtle seasonal differences in the phytoplankton response to N form were noted as well. For example, addition of urea had a marginal stimulatory effect on the dinoflagellate *G*. *instriatum* (Fig 1, Table 3), possibly due to low light conditions favoring mixotrophy over autotrophy (e.g., [42, 43, 65]). *G*. *instriatum* has been shown to bloom in other systems including the Chesapeake Bay [66], and winter-spring dinoflagellate blooms may contribute a substantial fraction of annual primary production in estuarine systems (e.g., NRE [33], Patuxent River Estuary [67]). Thus future work should consider seasonal light availability as a factor that may influence the relative importance of inorganic versus organic nutrients on growth of relevant dinoflagellate taxa. Furthermore, the potential for organic N availability (i.e., urea) to influence overall phytoplankton community structure and bloom dynamics during this time of year warrants further investigation. Another example comes from N-limited summer months, when addition of both forms of N stimulated phytoplankton growth to a similar degree. This suggests that urea supports growth across all investigated phytoplankton groups, likely due to mixotrophic capabilities of some taxa as well as rapid bacterial conversion of urea to ammonium and its subsequent uptake by other phytoplankton taxa (e.g, [68]). Overall, these findings reinforce current understanding of the effects of N pulses on estuarine phytoplankton communities, and add to a very limited body of knowledge on the relative effects of urea and nitrate on estuarine phytoplankton community growth and compositional responses.

Finally, our results stress the importance of continued use of microscopic identification/enumeration in phytoplankton research. While methods such as HPLC allow high throughput of data and broad descriptions of phytoplankton groups, results may obscure ecological dynamics due to marker pigment overlap between a wide range of taxa [34]. Additionally, cellular pigment concentrations can vary between strains as well as due to environmental variability (e.g., [57–59]). In some cases, this may lead to divergence in the relationship between pigment concentration and cellular abundance. Thus, employment of both targeted microscopy-based cell enumeration concurrent with pigment-based technology may be warranted in certain situations to facilitate a more complete understanding of phytoplankton dynamics in estuarine systems.

## Supporting Information

S1 Table Nutrient concentrations (μg L^-1^) for each experiment. (XLSX)

S2 Table Pigment concentrations (μg L^-1^) for each experiment. BDL indicates below detection limits.
(XLSX)

S3 Table Select phytoplankton taxa abundances (cells mL^-1^) for each experiment. (XLSX)
